# Self-Organization in High-Density Bacterial Colonies: Efficient Crowd Control

**DOI:** 10.1371/journal.pbio.0050302

**Published:** 2007-10-30

**Authors:** HoJung Cho, Henrik Jönsson, Kyle Campbell, Pontus Melke, Joshua W Williams, Bruno Jedynak, Ann M Stevens, Alex Groisman, Andre Levchenko

**Affiliations:** 1 Department of Biomedical Engineering, The Johns Hopkins University, Baltimore, Maryland, United States of America; 2 Department of Theoretical Physics, Lund University, Lund, Sweden; 3 Department of Physics, University of California San Diego, La Jolla, California, United States of America; 4 Department of Biological Sciences, Virginia Polytechnic Institute and State University, Blacksburg, Virginia, United States of America; 5 Center for Imaging Science, The Johns Hopkins University, Baltimore, Maryland, United States of America; University of Rochester, United States of America

## Abstract

Colonies of bacterial cells can display complex collective dynamics, frequently culminating in the formation of biofilms and other ordered super-structures. Recent studies suggest that to cope with local environmental challenges, bacterial cells can actively seek out small chambers or cavities and assemble there, engaging in quorum sensing behavior. By using a novel microfluidic device, we showed that within chambers of distinct shapes and sizes allowing continuous cell escape, bacterial colonies can gradually self-organize. The directions of orientation of cells, their growth, and collective motion are mutually correlated and dictated by the chamber walls and locations of chamber exits. The ultimate highly organized steady state is conducive to a more-organized escape of cells from the chambers and increased access of nutrients into and evacuation of waste out of the colonies. Using a computational model, we suggest that the lengths of the cells might be optimized to maximize self-organization while minimizing the potential for stampede-like exit blockage**.** The self-organization described here may be crucial for the early stage of the organization of high-density bacterial colonies populating small, physically confined growth niches. It suggests that this phenomenon can play a critical role in bacterial biofilm initiation and development of other complex multicellular bacterial super-structures, including those implicated in infectious diseases.

## Author Summary

Bacterial cells form colonies with complex organization (aka biofilms), particularly in response to hostile environmental conditions. Recent studies have shown that biofilm development occurs when bacterial cells seek out small cavities and populate them at high densities. However, bacteria in cavities may suffer from poor nutrient supply or waste removal, or disorganized expansion leading to blockage of cell escape. In this study, we observed Escherichia coli in a microfluidic device that allows direct observation of the growth and development of cell colonies in microchambers of different shapes and sizes through multiple generations. Combining this experimentation with computational analysis of colony growth and expansion, we characterize a process of colony self-organization that results in a high degree of correlation between the directions of cell orientation and growth of collective cell movement. We also find that this self-organization can significantly facilitate efficient escape of cells from the confines of cavities where they reside, while improving the access of nutrients into the colony interior. Finally, we suggest that the aspect ratio of the shape of E. coli and other similar bacteria might be generally subject to a constraint related to colony self-organization.

## Abstract 3

In nature, bacteria often found themselves in high-density colonies. The combination of a novel microfluidic device and computational analysis reveals an unexpected self-organization behavior of tightly packed bacterial cells.

## Introduction

The past few decades witnessed an emergence of the realization that bacterial cells in their natural environments are not asocial, but can exist as colonies with complex organization and exhibit sophisticated and highly regulated collective behaviors [1–5]. Consequently, significant efforts have been made to investigate the collective behavior of bacteria in various settings, with a particular emphasis on the formation of highly organized, multicellular super-structures. Instances of such colony formation include tightly packed bacterial “pods” in epithelial cells, colonies of luminescent bacteria in light organs of marine animals, or biofilms forming on plastic or glass surfaces in various high-humidity environments [6–10]. One important aspect of these naturally occurring tightly packed bacterial colonies (henceforth referred generically to as biofilms) is that they frequently arise despite, and possibly in response to, unfavorable environmental conditions including various types of chemical stress, variable temperature, fluid flow, the host immune system, and limited supply of nutrients [5]. In the initial stages of the biofilm development, it is crucial for bacterial cells to overcome the above-mentioned adverse environmental conditions, while laying foundations for highly ordered, mature biofilm structures. Recent studies have revealed that one of the important initial steps in this process might be for bacterial cells to actively seek out small cavities and populate them, reaching very high densities [11–13]. In addition to providing partial shelter, physical confinement might facilitate the onset of quorum sensing that is thought to be important for the successful progression of biofilm development. However, there are also severe potential disadvantages to forming packed colonies that are partially isolated from the surrounding environment, including increasingly poor nutrient supply and waste removal, as well as the possibility of disorganized expansion leading to cell damage and even blockage of cell escape from the growth niches. How cells cope with these constraints to successfully initiate biofilm development is currently unclear.

A clue to understanding cell behavior in these early stages of biofilm development might come from the high degree of multicellular organization found in stalk formation of yeast cells emerging from microscopic pits in agar gels [14,15]. Initial confinement of cells to small cavities and mechanical interaction between cells and the cavity walls appeared to be essential not only for the formation of complex tall structures uncharacteristic of lab yeast strains, but also for the high degree of functional order and differentiation within these structures. Colony organizations in various organisms such as Bacillus subtilis and Escherichia coli, that were visualized using scanning and transmission electron microscopy [2], also show strikingly well-ordered multicellular arrays within the colonies. Recently, with the aid of advanced microscopy systems, biofilm structures were also found to comprise millions of bacterial cells in regular arrangements that facilitate the physiological interactions within the community [9]. It is of interest therefore to investigate whether this functional organization in biofilms, as in yeast stalks, might emerge from the initial ordering of bacterial colonies growing in small cavities—ordering that might facilitate nutrient exchange and relieve the mechanical stress stemming from cell proliferation.

Although these dynamical reorganizations of the biofilm-like bacterial colonies are likely to be controlled by cell–cell interactions, lack of convenient experimental platforms allowing a dynamic analysis of large, tightly packed colonies on the single cell level has hampered research progress in this area. Recently, we developed a microfluidic device with chemostatic microchambers, which allows bacterial cells to grow over multiple generations in controlled microenvironments [16]. However, these chambers were relatively deep (∼6 μm), making it difficult to reach single-cell resolution at high cell densities. In addition, the device was designed to prevent the escape of cells from the chambers, and the packed colonies could only be monitored over a very limited number of cell generations. In this study, we sought to overcome these limitations and create a device for monitoring tightly packed colonies of actively dividing cells in chambers with different geometries over at least 24 h with single-cell resolution. Using a combination of modeling and experiments in these new devices, we found that developing E. coli colonies achieve progressively higher levels of spatial organization, enabling them to increase the nutrient supply and the efficiency of escape from the chamber. The results of the study suggest added importance of the asymmetrical shape of some bacterial species and have direct implications for biofilm organization. The device we describe can also have wider applications for robust long-term culture of bacterial cells under controlled conditions with real-time microscopy at single-cell resolution.

## Results

### The Design of the Microfludic Device

**Figure 1:**
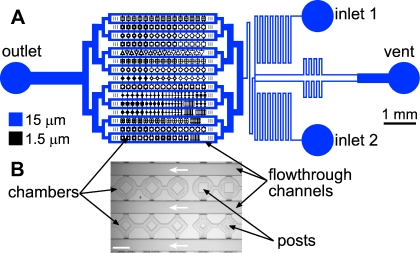
Microfluidic Device (A) A drawing of the microchannels; microchannels with depths of 15 and 1.5 μm are shown in blue and black. (B) A micrograph of a fragment of the array of channels and chambers. White arrows indicate the direction of flow. Scale bar, 100 μm. See a magnified view of the central part of the device in Figure P1 of Protocol S1.

Similar to the previously described microfluidic chemostat [16], the main functional area of the microfludic device (Figure 1) is an array of flow-through channels and chambers between the channels. The symmetric binary branching of the flow-through channels from a single channel on both the inlet and outlet sides leads to highly balanced pressures at opposite sides of the chambers. Because the depth of the chambers is much smaller than the depth of the flow-through channels (∼1.5 μm versus 15 μm; Figure 1A), the chambers are much more resistant to flow than the channels. Therefore, there is practically no active flow through the chambers (flow at 0.1–0.2 μm/s for a typical channel flow rate of ∼100 μm/s), and the exchange of chemicals between them and the flow-through channels occurs by diffusion only.

There are three important differences between the device in Figure 1 and that described in [16]. First, the depth of the chambers of our device is close to the diameter of E. coli cells (∼1 μm) and substantially smaller than the cell length (∼3 μm). Therefore, all cells in the chambers are oriented in the plane of the device and create a monolayer, making it possible to detect cellular responses at a single-cell resolution. Second, there are no capillaries impermeable for cells between the flow-through channels and the chambers. Therefore, there are no physical barriers for cells to enter and exit the chambers. Third, although the distance between all flow-through channels is the same (140 μm), the chambers greatly vary in shapes (Figure 1B). The shapes of the outer boundaries of the chambers include circles, squares, diamonds, triangles, and strips. Furthermore, many chambers have one or several posts inside, adding inner boundaries to the geometries (Figure 1B). The posts have different sizes and are shaped as circles, squares, diamonds, triangles, parallel rectangles, and crosses.

### The Development of a Microcolony in a Confining Space

As in the cells in a microfluidic chemostat [16], the E. coli cells loaded into the chambers were found to readily form microcolonies. The microcolonies started from as few as 1–2 cells and eventually filled the chambers completely, with cells being in a densely packed state. However, since cells could freely exit the chambers into the adjacent flow-through channels, cell proliferation was not limited in time and continued for as long as the flow of fresh medium in the flow-through channels was maintained. The combination of cell proliferation and escape led to a continuous collective motion of cells of the densely packed colonies toward the microchamber exits. The rate of flux of cells through the exits is proportional to the rate of colony expansion and, given constant volume of the chamber and constant number of cells in the colony in a steady state, the rate of flux is also proportional to the mean cell growth rate in the colony. We did not observe any noticeable decrease in the growth rates of high-density colonies during prolonged continuous incubation, as judged by the characteristic rate of motion of cells toward chamber exits (see, e.g., Figure S3A).

For effective visualization of the organization of microcolonies, we used a strain of *E. coli*, transformed with a low-copy plasmid carrying green fluorescent protein (GFP) controlled by the truncated version of the *Vibrio fischeri lux* operon responsive to exogenously added *V. fischeri–*specific autoinducer [17], N-3-oxo-hexanoyl homoserine lactone (HSL), but deficient in endogenous AI production (Figure S1). In the presence of exogenously added autoinducer supplied with the medium ([AI] = 10 nM), the GFP mediated fluorescence allowed us to visually identify individual cells and perform analysis of their shape, size, and orientation.

**Figure 2:**
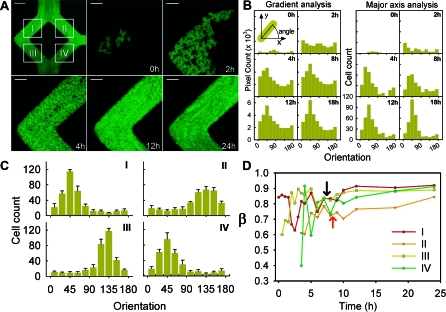
Self-Organization of an E. coli Colony in an RIR Chamber (A) Representative time-lapse epi-fluorescence images of an *E. coli* colony expressing GFP under the LuxR-*lux* box control. The fluorescence intensity was adjusted for visualizing the colony at single-cell resolution. The first sub-panel is a large-scale view of the RIR chamber, and the other sub-panels show the left “elbow” region of the chamber at different time points (0,2,4,12,24h). Scale bars: 30 μm, the 1st sub-panel; 10μm, all other sub-panels. See also Video S1–S3 (B) Comparison between the gradient and major axis analyses of cell orientation. Histograms of cell orientations in region I in (A) resulting from each analysis are shown for indicated time points. The range of the *x*-axis is in degrees in all histograms in this and other Figures. (C) The steady-state orientation histograms for cells in regions I–IV. The frequency within each bin is averaged over five time points between 19 and 24 h. Values are expressed as means ± standard deviation. (D) Time-dependence of the fraction, β, of cell population oriented within 45° of the preferred cell orientation in selected regions. The preferred cell orientations were estimated based on the steady state orientation distributions: I- 45°; II- 135°; III- 135°; IV- 45° with respect to the *x*-axis. The arrows indicate when the colony becomes densely packed (black: I–III; red: IV).

We analyzed images of the colonies in different chambers (see Videos S1–S8 for typical examples) and were surprised to observe that, at high densities, the orientations of cells were often anisotropic and highly correlated over distances much larger than the cell length. Typical evolution of a colony in one of the chambers is shown in Figure 2A. Both inner and outer boundaries of the chamber are shaped as rhombi (“rhombus in a rhombus” or RIR chamber). The cell growth initiated in region I, and the orientations of cells appeared random in the beginning. The microcolony then filled the entire chamber to dense cell packing in 8–9 h of growth. (Dense packing in a subcolony was defined as a state in which there was no cell-free region with the area equal to or greater than the area of a daughter cell following a cell division.) As the colony reached dense packing, most cells gradually became oriented along the direction of the collective cell flow toward the chamber exits, which frequently coincided with the directions of the internal and external walls of the chamber.

To quantify the orientation of cells, we processed fluorescence images of the chambers using two different methods that we termed the “gradient analysis” and the “major-axis analysis” (Protocol S1 and Figure S2). In the major-axis analysis, the images were segmented to outline individual cells. Following this, the orientations of the major axes of the cells were determined. In the gradient analysis, cell orientation was estimated by determining the direction of the fluorescence gradients from the pixel intensity matrices, without explicit identification of individual cells. Sharp changes of fluorescence occur at cell boundaries, and the directions of fluorescence gradients are perpendicular to the orientation of cells. We applied both methods to sequences of images taken with an interval of 1 h in selected regions of the chambers and found that the two methods consistently yielded similar results (Figure S2).

In region I of the RIR chamber (Figure 2A), at the early time points 0 h and 2 h, the orientation histograms had nearly uniform distributions, indicating that cells were oriented randomly (Figure 2B). As the cell density in the colony increased, the distribution of cell orientations evolved to a shape with a peak at 45°, the direction of the chamber walls and of the collective cell flow in this region. Histograms of the orientation of cells in densely packed states in the other selected regions of the chamber (Figure 2A) were similar to the distribution in region I (Figure 2C). In particular, all histograms had peaks at angles corresponding to directions of the chamber walls—135°, 135°, and 45° for regions II, III, and IV, respectively. The shapes of the orientation histograms of densely packed colonies at distinct time points differed relatively little (Figure 2C), and there were no detectable tendencies in the variation of their shapes with time (Figure 2B). This invariance of histograms indicated that the microcolony reached a steady state in terms of cell orientation.

To assess the evolution of cell orientation, we introduced an order parameter, β, calculated as the fraction of cells oriented within ±45° of the angle at the peak of the eventual steady state histogram (e.g., at 0°−90° for region I). We plotted the time evolution of β for the four regions of the chamber (Figure 2D). The value of β is 0.5 for randomly oriented cells and it is 1 when all cells are aligned in the preferred direction, which is parallel to the chamber walls. Values of β ∼ 0.5 with large variations in time, suggesting basically random orientation of cells with large fluctuations, were typical at the early stages of colony development when cell densities were low. As time progressed and cell densities increased, variations of β became minimal, as expected for a microcolony at a steady state. In this steady-state regime, the values of β were 0.85 on average, indicating a major bias in the orientation of cells toward the directions of the nearby chamber walls. (Calculation of the order parameter using a reduced range of angles around the preferred orientation resulted in smaller values of β at the steady state but did not change the shapes of the curves.) The large value of the order parameter was attained in spite of the fact that the distance between the walls (∼30 μm) was much larger than the cell diameter. The cells in the region I reached a highly ordered state earlier, and in the region II reached it later, than cells in other regions, in accordance with the relative timing of expansion of the colony into these regions.

**Figure 3:**
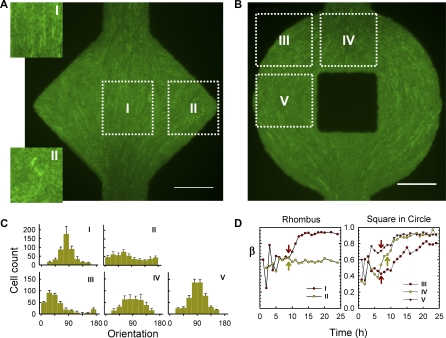
Colony Self-Organization in Chambers of Different Geometries Colony self-organization in various regions in the chambers of two geometries: rhombus (A) and square in circle (B). The images of the chambers were obtained in the same experiment as in Figure 1 (Videos S4–S8). (C) The histograms of the steady state cell orientations in the selected regions based on major axis analysis as in Figure 1. (D) Time-dependence of the fraction, β, of cell population oriented within 45° of the preferred cell orientation in selected regions: I- 90°; II- 90°; III- 45°; IV- 90; V- 90° with respect to *x*-axis. The arrows indicate when the colony becomes densely packed. Scale bars: 50 μm.

In addition to the RIR chamber, we used the same methods to analyze the cell orientation in chambers with other shapes. Two typical examples are described here. One chamber was shaped as a rhombus with no internal boundary (Figure 3A), and the other was shaped as a circle with a square internal boundary (Figure 3B). In both chambers, the walls were substantially further apart from each other than in the RIR chamber, which could be expected to lead to less order in the orientation of cells. Surprisingly, the analysis of images of densely packed colonies in both chambers showed that, in most regions, the orientation of cells was highly correlated over large distances. In a chosen region of interest (e.g., regions I, III, IV, and V in Figure 3A and 3B), the preferred orientation of cells usually coincided with the orientation of a nearby wall and always coincided with the direction of flow of cells toward chamber exits. In the central region of the rhombus-shaped chamber (region I and inset I in Figure 3A), close to 95% of cells were oriented within ±45° from the vertical axis, which was the direction of flow toward the chamber exits (Figure 3C and 3D). The orientations of cells in region I were highly correlated over a distance ∼20 times the cell length, in spite of the absence of chamber walls nearby. The orientation order was similarly high in most other regions of the chamber. Exceptions were the side corners (region II and inset II in Figure 3A), where cells appeared to be oriented nearly randomly, as indicated by a close-to-uniform distribution of angles (Figure 3C) and the order parameter β ≈ 0.5 in the steady-state regime. In the “square within a circle” chamber, the distributions of cell orientations in regions IV and V reached the steady states rapidly, and the order parameter attained high values β *>* 0.9 (Figure 3C and 3D). In region III, the steady state was reached considerably later than in regions IV and V, and the cell orientation was less anisotropic, as indicated by a lower value of the order parameter, β ≈ 0.8 (Figure 3C and 3D). In contrast to regions III and V, in region IV, the direction of flow of cells toward the exit (the flow direction defined the preferred orientation of cells) was orthogonal to the orientation of a nearby chamber wall. As a result, the flow pattern in region IV was rather complicated, with two fluxes of cells (from the left and from the right in Figure 3B) merging into one and with an analogue of a separation line in the middle.

### Simulations of the Microcolony Development

**Figure 4:**
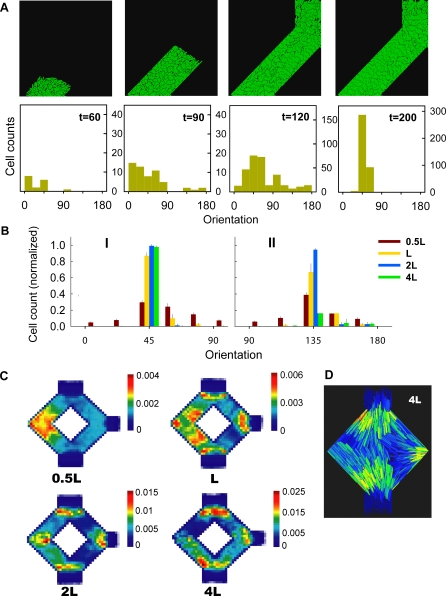
Computational Modeling of Colony Self-Organization (A) The organization of a colony and the corresponding distributions of cell orientation based on the major axis analysis of the simulated data at four different stages of the colony development within the region I of the RIR geometry (see Videos S9 and S10 for the RIR and S11 for a rhombus microchamber). (B) Comparison of the cell orientations from simulations with different cell lengths in quadrants I and II of the RIR geometry (0.5*L*, *L*, 2*L*, and 4*L*; see text and Videos S12–S15 for all the quadrants). To aid the visual comparison, histograms are shown in the ranges where the majority (90%) of cells are found. Each bar represents the average value calculated from 200 simulation time points, all in a densely packed state; the error bars indicate standard deviation. (C) Simulated spatial distributions of the force metric for different cell lengths. Simulations following dense packing were used to calculate average spatial distributions of mechanical stress experienced by cells (see Protocol S1 for definition of the force metric; zero stress corresponds to virtually no force experienced by the cell; see Videos S12–S15 for the distributions at single-cell resolution). (D) A snapshot of a simulated colony of the 4*L* cells at high density. The mechanical stress experienced by individual cells is color-coded. See also Videos S16.

The observation that a high degree of correlation in cell orientation occurred only at high cell densities, in the absence of active cell movement, suggested that the correlation originated from purely mechanical forces. Locally, these forces result from contact interactions of cells with each other and from the constraint of 2-D motion in the plane of the chamber. Globally, the cells are mechanically influenced by their collective motion toward chamber exits. The pattern of this motion depends on the position and orientation of the chamber walls and exits. To investigate if cell self-organization could be solely due to these contact interactions, we simulated the development of a colony in a 2-D region corresponding to the footprint of a chamber, with cells modeled by 2-D objects with shapes, lengths, and widths typical for E. coli cells. The model simulations were based on our previously reported analysis of yeast colony growth [11] with appropriate modifications accounting for differences in cell shape, for symmetric cell division, for the presence of boundaries, etc. (see Methods for more details). Cells were assumed to grow steadily along their major axes, to divide at even time intervals, and to have spring-like deformation potentials. Forces experienced by cells were due to cell–cell and cell–wall interactions. We found that this simple mechanical model reproduced all the major features of colony expansion in different geometries, including the gradual onset of anisotropic orientation of cells with long-range spatial correlations at high cell densities. Both the time evolution and the steady-state distributions of the cell orientation angles in the RIR chamber strongly resembled the corresponding experimental results (Figures 2 and 4A). Similar evolution and steady-state patterns were obtained in the other modeled geometries (Videos S9–S11 and unpublished data). The agreement between the model and experiments corroborated the suggestion that the self-organization of cells within the colonies originated from purely mechanical effects.

Since a preferred direction of orientation can only exist for nonspherical, elongated objects, the self-organization of E. coli cells in the colony and the anisotropy in their orientation are closely related to the elongated shape of E. coli cells. We used the model to examine how the orientation anisotropy and β in the high-density steady states depend on the ratio between the length and width of cells and simulated colonies composed of cells with maximum lengths shorter (0.5*L*) and longer (2*L*, 4*L*) than “normal” (the normal length, *L*, corresponded to the average aspect ratio 3.75:1 calculated given that the cell lengths vary from half-maximal to maximal in the course of cell growth). We found that cells with the reduced aspect ratio had smaller orientation order and lower β than the “normal” cells (0.81 vs. 0.99 for region I). On the other hand, we found no significant difference in the anisotropy of orientation between the “normal” cells and the cells with the increased aspect ratios, with the β values of 1.00 and 0.97 for 2*L* and 4*L* cells versus 0.99 for the normal cells. The results of the simulation suggest that the actual aspect ratio of E. coli (3:1–4:1) might be close to the minimal aspect ratio sufficient to ensure a high level of coordination of cell orientation within a colony.

We further interrogated the model to evaluate the forces experienced by cells of different lengths at different stages of colony development (see Methods, Videos S12–S15). For cells of 0.5*L* length simulated within a RIR chamber, the stress at the steady state increased with the distance from the chamber exits and was maximal along the horizontal axis of symmetry of the chamber (Figure 4C). The distribution of stress in the simulated colony was similar to the distribution of pressure in a model of a Newtonian fluid with spatially uniform source term, corresponding to a uniform and steady growth of cells in a chamber of the same geometry (Figure S3B).

By contrast, for the longer cells(2*L* and 4*L*) than “normal” cells, the simulations indicated the existence of areas of high stress near the chamber exits. Near the exits, the directions of flow of cells in two merging streams change abruptly by 45°, the total width of the cell stream decreases, and the flow lines merge, causing many cells to become misaligned. The misalignment can lead to high stresses due to both growth of cells in the direction perpendicular to the flow and possible “stampede”-like exit blockage exacerbated by the convergence of flow lines (Videos S13 and S14).

Additionally, we observed that if the cells were oriented perpendicularly to a nearby chamber wall and their growth was not directed toward one of the exits, the cells experienced high localized forces (Figure 4D, left and right corners of the chamber). This observation suggested that self-organization of colony expansion in parallel to the chamber walls may decrease the mechanical stress induced by cell growth.

### Facilitation of Nutrient Transport in Organized Colonies

A high degree of anisotropy achieved in the steady-state colonies might affect the efficiency of diffusive transport of nutrients into and metabolites out of the bulk of the colony through the chamber exits. Indeed, a recent theoretical study suggested that increased anisotropy of a porous tissue can lead to a dramatic reduction of its tortuosity and enhancement of diffusion in a preferred direction [18]. The study also analyzed diffusion in a 2-D medium with an array of excluded regions having shapes of identical extended rectangles, oriented parallel to each other, which is a good approximation of the high-density steady-state colonies in the microchambers. The effective diffusion coefficient in the direction along the rectangles was found to be 
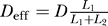
 , where *D* is the diffusion coefficient of the medium without excluded regions, *L*_1_ is the longer, and *L*_2_ is the shorter axes of the rectangles. For E. coli cells with the aspect ratios 3:1–4:1, in a highly ordered steady state with β ≈ 1, one might thus expect an effective diffusion coefficient of 0.75*D* to 0.8*D* along the directions from the chamber's exit into the bulk of the colony. For randomly oriented cells, *D*_eff_ would drop to 0.5*D* or even less, due to possible “dead-end pores”, i.e., blockages of openings between pairs of parallel cells by perpendicularly oriented cells. Thus, increasing the anisotropy of cell orientation within a colony is expected to enhance the supply of nutrients to internal regions of the colony and the evacuation of metabolites from the internal regions.

To test this prediction, we took advantage of the sensitivity of the *lux* operon to glucose levels. Transcription in this operon is positively regulated by the cAMP-receptor protein (CRP) [19], which is progressively activated at decreasing glucose levels due to elevated intracellular cAMP concentrations. As a result, the level of GFP expression substantially increased at reduced glucose levels that were expected to be found in densely populated microfluidic chambers with impaired diffusive transport (Figure P3 of the Protocol S1). We found that, for all chamber shapes, fluorescence of individual cells increased as the colony became denser and filled the chamber, which was consistent with the expected reduction of glucose concentration. Moreover, in the high-density steady-state colonies, well-formed gradients of fluorescence were often observed, with cells becoming progressively less fluorescent toward the chamber exits (Figures 2 and 3 and Figure S5). Because the exits were a source of the exogenously added autoinducer, this type of distribution of fluorescence in the colony was inconsistent with the possibility that autoinducer did not reach cells in the internal regions. The variation of the glucose level appeared to be the only plausible explanation of this fluorescence distribution, and we concluded that the level of cell fluorescence could be used as an indicator of the local concentration of glucose. This conclusion was further corroborated by a high degree of correlation between the expression of GFP driven by the *lux* operon promoter and the expression of HcRed protein under exclusive control of cAMP-CRP complex (both expressed in the same strain of E. coli) in different cells growing in the same chamber [20] (Figure P4 of Protocol S1).

**Figure 5:**
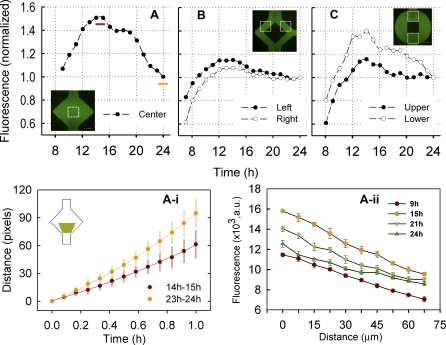
Glucose-Dependent *lux* Operon-GFP Response in Microchambers with Different Shapes: Rhombus (A), RIR (B), and Square in a Circle (C) The average fluorescence intensities in the selected regions were normalized to their value at the end of the experiment, which was taken as 1. (A-i) the flow of cells from the RIR chamber toward an exit during two time intervals corresponding to the red and orange bars in (A). An average of distances traveled by 10 tracked cells from their initial positions is shown as a function of time. The tracking was done in the region indicated by the green trapezium in the inset. The error bars are standard deviation. Each plot was fitted to a quadratic curve. (A-ii) the fluorescence intensity within the rhombus chamber was measured at indicated times along a line from the center (0 μm) to an exit (65 μm). Note the correspondence of the variation in the absolute intensity at distance 0 μm with the temporal variation shown in (A). Each data point represents the mean fluorescence value ± standard deviation from square regions containing approximately 30 cells each.

We then investigated the evolution of GFP fluorescence in different regions of the previously analyzed chambers (Figure 5). In the case corresponding to region I in Figure 3A (rhombus-shaped chamber), we found that, following a maximum level reached around 13 h, the fluorescence intensity gradually dropped by approximately 30% (Figure 5A). Additionally, the fluorescence intensity gradient along a line connecting region I with a chamber exit gradually became shallower, indicating better penetration of the nutrients into internal regions of the colony (Figure 5A-ii). These observations could be explained by noting that the alignment of cells in the rhombus chamber reached its highest level at least 4 h after the colony became densely packed. Therefore, initially, nutrient transport through the colony would be hampered by decreasing intercellular spaces and increasing nutrient consumption leading to increased fluorescence intensity. However, over time, the transport efficiency can be improved by increased anisotropy within the colony, leading to a progressive decrease in the GFP signal at the colony interior. Consistent with this hypothesis, the drops in the GFP fluorescence intensity from the maximal levels were much more limited in the other two chambers analyzed, where by the time the colonies became densely packed, cells were already arranged in a highly anisotropic fashion (Figure 5B and 5C). Taken together, the results suggest that fluctuations in the nutrient supply available to the colony can depend on whether the colony self-organization accompanies or follows achieving the densely packed state. A significant increase in the colony growth rate, as judged by the rate of cell movement out of a chamber, which was observed in the rhombus but not RIR chamber at a later stage of experiment provided independent further support for this suggestion (Figure 5A-i and Figure S3A).

## Discussion

This study has demonstrated that long-term growth of E. coli colonies within small enclosed spaces can be accompanied by self-organization of the colonies into states characterized by highly correlated cell orientation. A simple agent-based model incorporating the details of asymmetric cell shape, mechanical interactions arising from cell growth and division, and the confinement of colonies within specific boundaries has reproduced the salient features of this organization process. The success of the model strongly suggests that self-organization is due to localized mechanical cell–cell and cell–chamber wall interactions, and most importantly, elongated cell shape.

When viewed on a coarse scale—that of hundreds of cells—the dynamics of steady-state colonies bear a marked similarity to the patterns of flow of Newtonian fluid continuously generated within confined spaces of various shapes. This similarity is exemplified by the coincidence of the streamlines of Newtonian fluid with the directions of the collective cell motion (Figure S3B). In addition, the areas corresponding to high fluid pressure overlap with those of increased stress experienced by cells with low aspect ratio (Figure S3B). These simple patterns are further refined on the scales comparable to the size of a single cell, where the effects of elongated shape of the cells become especially pronounced. In particular, it becomes evident that not only the direction of movement but also the orientations of individual cells become aligned with the predicted streamlines and also become mutually correlated. The directional correlation increases with increasing cell aspect ratio. This self-organization behavior, involving hundreds to thousands cells, contrasts with the previously reported transient local alignment of relatively small cell clusters ([21], Figure S4), and is strongly dependent on the geometry of the confining boundaries and chamber exits and on the direction of the collective cell flow (Figure 3D and Figure S5). Based on the results described here, we propose that colony self-organization can endow the constituent cells with at least two important advantages.

First, orientation of cell growth toward the exits connecting microchambers to external space can lead to the relaxation of the stress experienced by the cells due to constriction by the chamber boundaries. In a cell population lacking such an orientational order, cell growth toward chamber walls can create local foci of high stress (see e.g., Figure 4D). Interestingly, increasing the cell aspect ratios beyond the values comparable to those of the wild-type E. coli does not seem to affect the degree of self-organization, but can increase the cellular stresses at the chamber exits due to rapid changes in cell orientation. In this regard, it is interesting to contrast the collective behavior of bacterial cells to the self-driven movement of a large crowd in a confined space. The model in [22] suggests that a combination of a relatively narrow bottleneck coupled with self-propelled, panic-like movement of people within a confined space can easily result in a blockage of the exits, leading to potentially widespread injuries. Arguably, bacterial cells in tightly packed colonies populating small cavities or other confining environments might face similar stampede-like blockage challenges. Our results suggest, however, that the potential for “bacterial stampedes” arises only for relatively long cells, which have feature sizes comparable to the dimension of the cavity exit openings. Therefore, from the standpoint of stress relief, one can speak of an optimal cell length, such that cells are long enough to undergo robust self-organization, and yet short enough to avoid increased stress at the exits from a confined space, and potential blockage of cell escape. It can be proposed that these constraints on cell shape, in addition to the potential importance of elongated cell shape for cell motility and other functional responses [23], might have contributed to the evolutionary pressure defining the aspect ratios of cells in many extant bacterial species, in particular those that have morphology similar to E. coli and form biofilms.

The second advantage of anisotropic colony organization is the reduction of the tortuosity of the intercellular spaces progressively enhancing the diffusion of nutrients into the colony. Efficient diffusive transport of nutrients and metabolites is an important determinant of the well-being of bacterial cells within the bulk of the colony, ensuring sufficient energy supply for cell division and other functions. A corollary of this finding is that signal molecules might also diffuse more easily along the flow lines formed by the cells, thus introducing anisotropy into cell-cell communication, e.g., through quorum sensing.

Our findings provide a fresh perspective on possible mechanisms of emergence of the structurally complex biofilms and other types of multicellular super-structures. Although the order inherent in biofilms is clearly shaped by a complex interplay of multiple processes, including intercellular signaling [24] and regulated migration of cell sub-populations within the developing and differentiating colonies [25], this order and the chances of survival of bacterial biofilms might critically depend on the initial self-organization of cells during their anchorage and expansion within small cavities and other niches in complex growth substrata. The complex mechanical interactions between cells themselves and between cells and physical boundaries confining them, determined in large part by cell morphologies, can help create patterns of extensive organization and enhanced chemical transport, which can lay the foundation of complex functional multicellular ensembles. The understanding of these processes might help control biofilm growth, and ultimately facilitate treatment of the related diseases.

Finally, we note that the microchamber device presented here provides a convenient and effective way to observe bacterial cells over multiple generations and track the evolution of a colony at single-cell resolution. It enabled us to observe the individual cellular response in both fluorescence and phase-contrast microscopic settings. We envision that the flexibility of design and convenience of use will make this type of devices a platform of choice for many scientific and biotechnological applications.

## Methods

Details on strain construction and growth conditions are presented in the Protocol S1. Epi-fluorescence imaging of GFP expression is described in the Protocol S1. Design and fabrication of the microfluidic device are included in the Protocol S1.

### Image analysis.

In our study, we defined the “orientation” of a cell as the angle that the cell forms with respect to the *x*-axis of the Cartesian coordinates of each image (inset in Figure 2B). Its range is [0°, 180°]. The custom software package to determine the cell orientations was developed and implemented in Matlab 7.0.0., including the image-processing toolbox. Time-lapse fluorescence images in the 16-bit TIFF format indexed by location and time stamps were used as the input. In the package, two different routines were applied to find the orientation of individual cells. (Gradient analysis and Major axis analysis; see Protocol S1 for a detailed description).

### Modeling.

Individual cells are explicitly modeled as separate agents and are assumed to have an area enclosed by two semi-circles attached to opposite sides of a rectangle of constant width but variable length. Positions and lengths of the cells are defined by two generalized coordinates designating the centers of the semi-circles attached to the rectangle. The dynamics of the colonies is considered to be dominated by viscous friction, with velocity rather than acceleration being proportional to the force. See Protocol S1 and Videos S17 and S18 for details of the model set-up and simulations.

## Supporting Information

Figure S1 A Representative Map of the Plasmid pJWP01S Used for Visualization of *lux* Operon Expression pPROBE-*gfp*(tagless) with *lux*R-*lux*box-P*_luxI_* Inserted into the *Eco*RI-*Kpn*I Sites of Multiple Cloning Site. (659 KB TIFF)

Figure S2 Two Different Approaches for Analyzing the Cell Directions in the Colony (A) The major axis analysis consists of several steps: (Left) start with the raw image; (Middle) find the local maxima (blue) and short axes (green lines); (Right) connect the short axes to the long axes and filter out the wrong axes to generate the final major axis (yellow lines).
(B) The gradient analysis. (Left) Compute the gradient of the image; (Middle) threshold the norm of the gradient; (Right) calculate the angle of the gradient with respect to the *y*-axis to determine the principal orientations of cells.
(C) Histograms of cell orientations based on two analyses show similar distributions.
(2.8 MB TIFF)

Figure S3 Quantification of Cell Outflow from the RIR Chamber (A) Quantification of the flow of cells from the RIR chamber toward an exit in a densely packed colony. Three consecutive time intervals were selected, all at the stage of the steady-state colony growth. For each time interval, 10 individual cells were chosen from the inner side of one of the green-shaded regions in the chamber shown in the inset (closer to the center of the chamber), and their positions on consecutive fluorescence images were manually tracked every 5 min. After 1 h, most of the cells were found at the outer side of the region (closer to the exit of the chamber). A typical trajectory of the cells is indicated by the arrow in the inset. The mean distance traveled by cells from their initial positions is shown as a function of time. The error bars indicate standard deviation. Each plot was fitted to a quadratic curve. The pair-wise difference among the three fits was not significant (*p-*values of <0.05 were considered significant; *F*-test). The mean velocity of the escaping cells is a measure of the rate of colony growth in the corresponding time interval.
(B) The simulated flow stream lines (arrows) and pressure distribution (color-coded) inside the RIR chamber and rhombus chamber filled with a Newtonian fluid with spatially uniform source term, corresponding to a uniform and steady cell proliferation.
(4.7 MB TIFF)

Figure S4 Colony Expansion with No Confining Boundaries (A) Comparison of simulation and experimental results of colony growth with no boundaries. Scale bar is 12μm.
(B) Distributions of cell orientations from the final stage of the simulation and the experiment.
(3.3 MB TIFF)

Figure S5 The Effect of Internal Boundaries in Microchambers on Colony Organization The external boundaries of all chambers are identical and have a shape of a square, whereas the internal boundaries have different shapes. Representative examples and enlarged views of selected regions, a and b, are shown. The internal geometries are (1) rhombus; (2) circle; (3) square; (4) “equality sign”; and (5) no internal boundary.
(A) An enlarged view of cell escape points (the exit of chambers). The cells facing the bottom exits (boxes 'a' in panels 1–4), which are situated in the separation areas between fluxes from the right and left of the exits, are found to be less fluorescent than cell in the surrounding regions. This is likely because cells in the separation area do not arrive from internal regions of the chamber, but rather grow and divide in relative proximity of the exit. In contrast, the fluorescence in the “flux” areas reflects the history of exposure of cells to lower glucose concentrations in more internal regions of the chambers. This effect becomes increasingly pronounced from panel 1 to panel 4, as the variations in the shape of the internal boundary facing the exit (from a pointed apex of a rhombus, to a circle, to a square, to a horizontal line on top of two exits) create increasingly wide separation areas.
(B) The orientation of the internal boundaries strongly influences the direction of orientation of cells in the colony. Notice that in the case of the “equality sign” (panel 4), the horizontal orientation of the internal boundaries imposes horizontal orientation on cells in the middle of the chamber, whereas when no internal boundaries are present (panel 5), the cell orientation is vertical. Scale bars: 50 μm, upper panels; 20 μm, panels (a) and (b). For examples of cell orientations in chambers of other geometries, please consult the Levchenko lab website: http://rd.plos.org/pbio.0050302.
(7.3 MB TIFF)

Protocol S1 Details of Methods and Supplementary Analysis (1.6 MB DOC)

Video S1 The Colony Growth in an RIR chamber (2.7 MB MOV)

Video S2 Zoom-In on Region I from Video S1
(2.6 MB MOV)

Video S3 The Full-Size Version of Video S1
(1.8 MB MOV)

Video S4 The Colony Growth in a “Rhombus” Chamber (2.3 MB MOV)

Video S5 Zoom-in on the Center Region of Video S4
(2.8 MB MOV)

Video S6 The Full-Size Version of Video S4
(1.6 MB MOV)

Video S7 The Colony Growth in a “Square in a Circle” Chamber (2.1 MB MOV)

Video S8 The Full-Size Version of Video S7
(1.8 MB MOV)

Video S9 A Simulation of the Colony Growth in an RIR Chamber (8.1 MB MOV)

Video S10 Zoom-In View on Region I from Video S9
(8.2 MB MOV)

Video S11 A Simulation of the Colony Growth in a Rhombus Chamber (3.5 MB MOV)

Video S12 A Simulation of the Spatial Distribution of the Force Measure of 0.5*L* Cells in an RIR Chamber (3.7 MB MOV)

Video S13 A Simulation of the Spatial Distribution of the Force Measure of 1*L* Cells in an RIR Chamber (6.1 MB MOV)

Video S14 A Simulation of the Spatial Distribution of the Force Measure of 2*L* Cells in an RIR Chamber (6.1 MB MOV)

Video S15 A Simulation of Spatial Distribution of the Force Measure of 4*L* Cells in an RIR Chamber (6.3 MB MOV)

Video S16 A Simulation of Spatial Distribution of the Force Measure of 4*L* Cells in a Rhombus Chamber (1.1 MB MOV)

Video S17 Cell-Division Dynamics, Simulation of Cell Division over Three Generations (445 KB MOV)

Video S18 Simulation in the RIR Chamber Starting from a Chamber Filled with an Even Distribution of Randomly Oriented Cells (8.0 MB MOV)

## Abbreviations

2-D: two dimensional
GFP: green fluorescent protein
RIR: rhombus in rhombus
