# Supplementary material for: Self-Organization in High-Density Bacterial Colonies: Efficient Crowd Control
Source: PLoS Biol. 2007 Oct 30;5(11):e302. doi: 10.1371/journal.pbio.0050302 (PMC2043048; doi:10.1371/journal.pbio.0050302)
Supplement: Supplementary file 1 [file pbio.0050302.sd001.doc]

**SUPPLEMENTARY PROTOCOL S1**

**Supplementary methods**

**The design of the microfluidic device**

The layout of the microfluidic device (Fig. 1 of the main text and Fig. P1) is similar to that of the microfluidic chemostat [1]. The functional area of the device is an array of 16 parallel flow-through channels with a depth of 15 μm and 1.5 μm deep chambers between the channels. The device has two inlets, inlet 1 and inlet 2, an outlet and a vent. Under normal operational conditions, the inlet 2 and the vent are blocked, and the inlet 2 is pressurized above the outlet to generate a pressure-driven flow through the channels. The inlet 2 and the vent provide an option of quickly switching the medium fed to flow-through channels. The layout of the device, with symmetric binary branching of the 1.5 μm deep channels on both the inlet and outlet sides (Fig. 1), leads to highly balanced pressures at opposite sides of the chambers. In addition, because of their small depth, the chambers are much more resistant to flow than the flow-through channels. Therefore, there is practically no active flow though the chambers, and the exchange of chemicals between them and the flow-through channels occurs by diffusion only [1].

**Figure P1**. Drawing of a fragment of the array of test chambers and flow-through channels in the Fig. 1 of the main text, showing the layout and shapes of the individual test chambers. 15μm and 1.5μm deep channels are shown in blue and black.

**Fabrication of the microfluidic device**

The device was fabricated using the same general protocol as in Ref. [1]. A ~5 mm thick cast of PDMS (Sylgard 184, Dow Corning) was made using a lithographically fabricated master mold. The master mold was a 4” silicon wafer with a two-level micro-relief (1.5 μm and 15 μm) of a UV-curable epoxy (SU8 by MicroChem, Newton, MA). The PDMS cast was cut into individual chips, which were punched with a gauge 20 lure stub to make inlet and outlet holes. The chips were soaked for 1 hr in a 0.01 N solution of HCl at 80 °C (to make the PDMS more hydrophilic), hermetically sealed to #1.5 microscope cover glasses, and baked in a 150 ºC oven for 30 min to increase hardness and the Young’s modulus of PDMS.

**Strains and growth conditions**

A truncated quorum sensing *lux* operon from *V.fischeri* was expressed in *E.coli* to track the multigenerational colony growth. An *Eco*RI-*Kpn*I fragment containing *luxR*, the *lux*box, and *lux*I promoter region was inserted into the MCS of pPROBE’-*gfp*-tagless vector to create pJWP01S (Fig. S1). This construct was used to transform *E.coli* strain MG1655. Overexpression of genes and the *gfp-*tagless reporter occurs in the presence of exogenously added *V. fischeri* specific autoinducer, AI [2], N-3-oxo-hexanoyl homoserine lactone (HSL) at a constant level ([AI] = 10nM). Before the on-chip experiments, *E.coli* strain MG1655 was cultured in LB broth with kanamycin 50 µg/ml at 30°C for overnight and diluted 1:100 to the fresh medium and grown to an OD_600_ of 0.2-0.3. Loading the cells into the chambers was performed following a procedure similar to that described previously [1]. After cells were loaded into the chambers, LB medium containing 10nM of AI was introduced to the channels. The flow was driven by a difference in pressure of ~0.5 psi between the inlet and the outlet, which was created either hydrostatically or using compressed air.

**Epi-fluorescence imaging**

Video-microscopy was performed with 40x/0.75 objective and a monochrome Cascade 1K digital camera (Photometrics, Tucson, AZ) on a Nikon TE2000 fluorescence inverted microscope equipped with a computer-controlled motorized stage. The excitation wavelengths of the filter for detecting GFP expression (Chroma, Rockingham, VT) were 450-490nm; the emission wavelength was 525nm. At the beginning of an experiment, 7-10 chambers of different geometries, each containing 1-5 cells, were identified. Cell colonies in each of the selected chambers were imaged under fluorescence and phase contrast illumination every five minutes. Acquisition of the images was pre-programmed and synchronized with motion of the motorized stage, which moved between the selected chambers in a loop-like fashion. The captured images were processed in the Slidebook 4.1. (Imaging Sciences, Inc., Denver).

**Effect of *E.coli* MG1655 AI-2 signaling.** We tested the possibility that *lux* operon may be affected by AI-2 signaling in the host strain *E.coli* MG1655. We studied the effects of

adding S-adenosylhomocysteine (SAH; an AI-2 precursor) and 4-hydroxy-5-methyl-3(2*H*)-furanone (MHF; an AI-2 homologue) [3] to cell medium and found no statistically significant changes in the induction of the truncated quorum sensing *lux* operon used in our studies, compared to the basal levels (Fig. P2). Therefore, we disregarded the effect of *E.coli* AI-2 mediated signaling on *lux* mediated GFP expression, as reported in the main text.

**Figure P2**. The effect of MG1655 AI-2 signaling on GFP expression driven by *V.fischeri lux* operon. The levels induced by 1 mM of MHF and SAH are contrasted with the base level of expression and with strong expression induced by 10 nM of AI. *E.coli* strain was batch-cultured in LB medium with 1mM substrates for 7 hours. The mean GFP expression was measured and analyzed at a single cell level. Error bars denote the standard deviations for three independent tests.

**Response of the *lux* operon to glucose.** Previous studies reported high sensitivity of transcriptional regulation of the *lux* operon to cAMP-CRP, with very little quorum sensing response when the concentration of active CRP is low (see e.g., [4]). To directly test the dependence of activation of the truncated *lux* operon on the glucose levels in the medium, we measured the fluorescence of a large number of individual cells at various concentrations of glucose in the defined minimal medium (Fig. P3). We found that the expression of GFP under the *lux* box control gradually decreased as the glucose concentration increased in 0-20 mM range. Note that 0.5% (w/v) glucose concentration (~27mM) is used conventionally in *E.coli* minimal medium for growth unrestricted by carbon source. In all experiments described in the main text, we used LB medium, which is a rich medium containing an unknown concentration of glucose. Based on the level of response of the operon in liquid LB culture, we estimate glucose concentration to be at least 10-20 mM, but significantly decreasing in densely packed chambers.

**Figure P3.** The effect of extracellular glucose on *lux* promoter–driven GFP expression. GFP florescence intensity in single cells was measured during exponential growth phase. Cells were incubated in the minimal medium complemented with 10 nM AI and the indicated concentrations of the glucose for 7 hrs, transferred onto cover slips, imaged and analyzed with Slidebook (Intelligent Imaging) to determine fluorescence intensity per cell. Error bars denote the standard deviations of the mean fluorescence values for three independent tests.

**Validation of *lux* operon as glucose sensor for experiments in shallow chambers.**

To examine the accuracy of estimation of the glucose levels based on the *lux* operon mediated GFP expression, as described in the main text, we transformed into MG1655 strain a plasmid containing red fluorescence reporter (HcRed), whose expression is driven by cAMP-CRP complex-binding promoter and is independent of the presence of HSL signal (a gift from Oudenaarden’s lab, MIT) [5]. We then grew a mixed colony of this strain and MG1655 transformed with pJWP01S in a rhombus chamber (Fig. P4). We evaluated red and green fluorescence in adjacent locations during 9 hr out of a 21 hr long experiment. We found that HcRed expression driven by cAMP-CRP complex bound promoter showed excellent linear correlation with GFP expression driven by *lux* operon

**Figure P4**. *Left panel.* Two-color fluorescence micrograph of a rhombus microfluidic chamber with a colony containing cells from two strains, MG1655 with pJWP01S expressing GFP and MG1655 with HcRed-expressing plasmid. Scale bars are 15µm (main panel) and 50 µm (insert). *Right panel*. Mean HcRed fluorescence vs. mean GFP fluorescence in the adjacent regions (box 1 and 2) is plotted in arbitrary units in four different time intervals over 9 hours. Each data point shows fluorescence intensity in box 1 vs. box 2 at a particular time point. The fluorescence intensity values were strongly correlated throughout the entire time of measurements (correlation coefficient = 0.999334).

promoter in pJW01S (Fig. P4). We thus concluded that the truncated *lux* operon expressed from pJW01S was a faithful reporter of the local glucose concentration.

**Cell orientation analysis**

The first routine was developed to detect the cell orientation by performing 2D convolution of pixel intensity matrices. The output of the first routine was the corresponding set of the orientation matrices of the same size as the input image. The second routine was designed to use the shape of a cell to recognize the cell major axis and corresponding cell orientation. The output of the second routine was the corresponding set of data containing the positions of the start and end points and the angle of each cell’s major axis for each time frame. Two different approaches were used to find the orientation of individual cells in a growing colony.

A) ***Major axis approach***

Let the rod-shaped *E. coli* cell body be divided into two regions: the elongated rectangular body and two semi-circular tips. This geometry presupposes the existence of a well defined major axis of a cell (Fig. P5) that can be used to characterize the cell’s position, orientation and length. This axis is assumed to pass through the center of and be of the same length as the body of the cell. Here we describe the algorithm used for determination of the positions of major cell axes in a colony. Two estimates are required to initiate the algorithm: *l*min, the minimal length of the cell (usually the length of a daughter cell immediately after division; we did not notice any significant change in the cell length over the generations) and *hw*, the estimated half width of a cell. Although not required, the estimated maximal cell length will be denoted as *l*max below.

**Figure P5**. The schematic images of a single *E.coli* for A) axis analysis

The procedure for finding the major axes of cells consists of 3 steps:

1. *Localization of cell bodies* (Fig. S2A, left panel). We assume that in the input fluorescence image, after a proper smoothing filtering (e.g., Gaussian low pass filter in MATLAB Image Analysis toolbox), the local maxima fluorescence intensity are located in a cell body and also close to the corresponding major axis. Under most conditions, this assumption was well supported by independent image analysis. The positions of the local maxima are thus recorded and used for subsequent analysis.
2. *Determination of the direction of major axes* (Fig. S2A, middle panel). The fluorescence intensity values in a rectangular region with *l*min/2 in height and *hw* in width and terminating on each local maximum are sampled while rotating this rectangle around the local maximum. The rotation is parameterized with the directional angle *θ* with respect to the horizontal axis. The *θ* dependent objective function *f(θ)*) is defined as the total fluorescence intensity values of the points in the rectangular region. The optimization problem is to find *θ*max = argmax(*f(θ)*), with the resulting *θ*max defining the angle of the orientation of a part of the cell corresponding to the local maximum. Using the information regarding the local maxima and the corresponding *θ*max, a set of short axes (denoted as *sax*) is constructed with each *sax* having the following properties: a) it contains the local maximum point (its starting point), b) it extends according to the corresponding *θ*max by the length *l*min/2 (reaching its end point). A preliminary analysis showed that a single cell normally contains several local maxima. It is thus necessary to merge the corresponding *sax* segments into a single longer axis. By definition, if the shortest distance between two *sax* segments is less than 3 pixels and they have a similar orientation (the difference in the angles *θ*max is smaller than /6), they are merged by translating an arbitrarily chosen *sax* segment along the shortest distance line and rotating it, so that it becomes parallel to the second *sax*. This procedure leads to a set of long axes (denoted as *lax*).
3. *Finding the tips of the cells* (Fig. S2A, right panel).. This step completes approximation of both the direction and the length of a single cell. Each *lax* segment is extended in both directions so that the florescence intensity values of the pixels in the extended parts are within 20% of those at corresponding end points of the *lax* segment. The length of the new extended lines is saved and denoted as *elax*. Then, the *elax* set is adjusted to remove the ‘wrong’ *elax* segments found in two distinct cases. First, when two or more virtually parallel *elax* segments are found in a single cell, they can be merged following the strategy used for merging *sax* segments, but allowing for up to *hw* distance as the upper threshold for merging (this step often requires manual intervention to prevent merging of two *lax*s in adjacent cells) . Second, there are two intersecting *elaxs* (each corresponding to the same cell). In this case, the one with less *sax* nodes is removed. After filtering out the wrong *elaxs*, one constructs a new set of the reduced *elaxs* (denoted as *relax*), each approximating to the length of an individual cell.

By visual inspection, we determined that this algorithm can correctly identify the lengths and orientations of at least 90% of the cells of interest.

B) ***Gradient orientation approach***

In the first step, a two-dimensional convolution was implemented using Gaussian low pass filter followed by Sobel horizontal edge-emphasize filter (predefined in Matlab Image Analysis Toolbox) to extract the horizontal edges. Then transposing the Sobel filter extracted the vertical edges (Fig S2B. left panel). The horizontal and vertical edges were combined to calculate the gradient magnitude of each pixel in the image. After thresholding the gradient magnitude, the contours of the cell containing area were determined (Fig. S2B, middle panel). In the second step, the gradient orientation was calculated by determining the angle of the gradient with respect to the x-axis. Y-axis was oriented downward. The angle range was [-π/2, π/2]. From the resulting matrix, the angle that the orthogonal to the gradient made with x-axis was calculated. Y-axis was re-oriented upward. The range was converted to [0,180]. The calculated values estimated the principal orientations of individual cells (Fig. S2B, right panel).

**Modeling**

**Cell description.** Individual cells are modeled explicitly. In the two-dimensional (2D) simulations, a cell is represented by a rectangle with two half-circles fused to its opposite sides. All cells have the same radius,

**Figure P6**. The schematic images of a single *E.coli* for computational model

*r*, which is the half-width of the rectangle and the radius of the half-circles in the 2D simulation, and which remains constant as the cells grow and divide. The position of a cell is given by two generalized coordinates, ***x***_1_ and ***x***_2_, that are the coordinates of the centers of the two half-circles as shown in Fig. P6. The length of the cell, *L*_r_, is the difference (distance) between ***x***_1_ and ***x***_2_ (the length of the rectangle), when the cell is under no mechanical stress. The cell colonies are assumed to exhibit strongly viscous behavior and all inertial effects are neglected when considering the dynamics of the system [6,7]. The equations of motion are given by

$$\frac{{dx}_{1,2}^{(i)}}{\text{dt}}=\frac{1}{z}F_{1,2}^{(i)}\text{=-}\frac{1}{z}Ñ_{1,2}V^{(i)},$$

where  is the friction coefficient and $V^{(i)}=V_{\text{cc}}^{(i)}+V_{\text{cw}}^{(i)}+V_{\text{int}}^{(i)}$ is the potential for cell *i*. $V_{\text{cc}}^{(i)}$ defines the contributions from cell-cell interaction mechanics, $V_{\text{cw}}^{(i)}$ from cell-chamber wall interaction mechanics, and $V_{\text{int}}^{(i)}$ is an internal (spring-like) potential, which is zero when the actual cell length is equal to its relaxed length, *L*_r_. In addition to the equations of motion, cell growth and division rules are applied, as described below.

**Cell-cell interactions.** An excluded volume potential is used to approximate cell-cell interaction. Cells are treated as solid bodies, which interact mechanically with neighboring cells. The potential on a cell *i* from a neighboring cell *j* is proportional to *H*_ij_^5/2^ where *H_ij_* = *H_ij_*(***x***_1_^(i)^,***x***_2_^(i)^;***x***_1_^(j)^,***x***_2_^(j)^) is the overlap that cell *i* and cell *j* (with coordinates ***x***_1_^(i)^,***x***_2_^(i)^;***x***_1_^(j)^,***x***_2_^(j)^) would have had if there were no interaction between the cells and no cell deformation [8]. Consequently, the cell-cell potential, $V_{\text{cc}}^{(i)}$, for cell *i* with *N*^(i)^ neighbors is defined as

$$V_{\text{cc}}^{(i)}\text{=-}\frac{2}{5}k_{\text{cc}}\sum_{j=1}^{N^{(i)}} H_{\text{ij}}(x_{1}^{(i)},x_{2}^{(i)};x_{1}^{(j)},x_{2}^{(j)})^{5/2},$$

where the parameter *k*_cc_ is proportional to the resistance of cell to deformation and sets the strength of the interaction. The overlap function, *H_ij_*(***x***_1_^(i)^,***x***_2_^(i)^;***x***_1_^(j)^,***x***_2_^(j)^), can be expressed analytically by calculating the shortest distance, *d_ij_*, between the two line segments ***x***_2_^(i)^ - ***x***_1_^(i)^ and ***x***_2_^(j)^ -***x***_1_^(j)^ and defining *H_ij_* = *φ*(2*r* - *d_ij_*), where *φ*(*x*) is the piecewise linear ramp function, *φ*(*x*)=*x* if *x*>0 and *φ*(*x*)=0 if *x*0. The overlap is always assumed to be much smaller than *r*.

**Cell-chamber wall interactions.** A wall, in our simplified description, is defined as a stationary line segment. The cells interact with walls in the same way they interact with other cells, with the only differences being in the value of the interaction constant and in that the wall positions do not change in time. The cell-wall interaction contribution to the potential for cell *i*, $V_{\text{cw}}^{(i)}$, is given by

$$V_{\text{cw}}^{(i)}\text{=-}\frac{2}{5}k_{\text{cw}}\sum_{w} H_{\text{iw}}(x_{1}^{(i)},x_{2}^{(i)})^{5/2},$$

where *k*_cw_ sets the strength of the interaction and *H_iw_* is the overlap between cell *i* and wall *w*. The overlap is again defined by a piecewise linear ramp function based on the shortest distance between the cell and wall line segments, *H_iw_* = *φ*(*r* - *d_iw_*).

**Cell size, growth and division.** To connect the cell coordinates with the postulated cell length, an internal cell potential, $V_{\text{int}}^{(i)}$, is introduced as a spring potential given by

$$V_{\text{int}}^{(i)}=\frac{1}{2}k_{\text{int}}(\left| x_{2}^{(i)}-x_{1}^{(i)} \right|-L_{r})^{2},$$

where *k*_int_ is the spring constant, $\left| x_{2}^{(i)}-x_{1}^{(i)} \right|$ is the distance between $x_{1}^{(i)}$ and $x_{2}^{(i)}$ (the actual cell length), and *L*_r_ is the length of the cell under no stress. This formalism is related to using a Lagrange-multiplier for constraining coordinates to be at the cell length distance.

All growth is assumed to be along the symmetry axis connecting ***x***_1_ and ***x***_2_, whereas the radius, *r*, is held fixed. The cell growth is modeled by exponential growth of the relaxed length *L*_r_ , which is truncated at a maximal size [9], resulting in the dynamic equation

$$\frac{\text{dL}_{r}}{\text{dt}}=k_{g}L_{r}(1-{L_{r}}/{L_{\text{max}}})$$

where *k*_g_ is the growth rate and *L*_max_ is the maximum cell length (see Fig. P7 for the corresponding simulation and its comparison with the solutions to this equation).

**Figure P7**.Simulaited time evolution of the growth and proliferation of two bacterial cells shown for illustration. The analytical solutions to the growth equation (lines) are shown overlaid on the simulation data (points). Also see the corresponding Video S11.

Cell division in the model is triggered by the growth (size) of the cells and no underlying molecular mechanism is modeled. When cells reach a threshold length, *L*_Th_, they split into two cells at a certain position along the cellular axis, producing two new cells with no overlap. The sizes of the daughter cells are close to being equal, with a small random deviation that has an amplitude *p*_div_ (introduced to break the axial symmetry and model commonly observed small variations in the cell growth rates and the positions of cell division plane). Given a mother cell of length *L*, the two daughter cells will assume lengths 0.5*L* (1 ± *p*_div_*R )- r* , where *R* is a uniform random number in [0:1] and *r* is subtracted to account for the addition of two semi-circles at each of the newly forming cell ends.

In the model, it is assumed that the conditions are such that the cell growth rate does not vary between different parts of the microfluidic device or over time. The model and experiments can be compared by locally tuning the cell growth rate in the model to obtain the same average cell division time as observed in the experiments (it could be variable e.g., due to local depletion of nutrients). A much more detailed model could be constructed that would also take into account variations between individual cells, but we have chosen to keep the model simple, assuming that such small variations would not affect the major results of the simulations.

**Force metric**. The metric of the force experienced by a cell was the sum of squares of the forces calculated at ***x***_1_ and ***x***_2_. The force exerted on the cell at each of the two positions was calculated as the spatial derivative of the total potential $V^{(i)}$with respect to the given position. The results did not change qualitatively if the values of the interaction potential were used instead of the values of forces.

**Variation of initial conditions.** To examine robustness of the eventual cell self-organization with respect to perturbations in the initial conditions, a simulation of colony growth in a microfluidic device was initiated with randomly oriented cells evenly distributed over the model area at a moderately high density (Fig. P8). The simulation showed that the difference in the initial condition had little effect of the eventual steady state organization of the dense colony that was the same as reported in the main text.

**Figure P8.** Simulations in the model RIR chamber starting from a chamber filled with an even distribution of randomly oriented cells. The whole chamber and a zoomed-in view of the upper left region are shown for the initial and final time points of the simulation. See also Video S18.

**Lack of a large scale colony self-organization in the absence of confining boundares.** To address the question of whether the mutual cell alignment is expected under any circumstances, regardless of the presence of confining boundaries and exits, simulations of freely growing and expanding colonies were performed. We saw small clusters of locally aligned cells that transiently emerged and quickly dissipated, similar to what was found experimentally in previous studies [10,11], but the stable, more global alignment was never observed. Figure S4 depicts an example simulation, along with the corresponding angular distributions determined for the final time point, juxtaposed with the experimental data showing a very similar evolution of colony growth and expansion.

**Parameter values.** Values of all parameters used in the simulations are presented in Supplementary Table 1. Spring constants were chosen sufficiently large to ensure small overlaps between pairs of cells and between cells and walls at the cell growth rate used in the simulations. As a simple robustness check, different parameters were individually varied twofold (up and down), which resulted in only minor changes in measured behavior except for when varying the cell size parameter *L_max_*. All major results have also been re-iterated using different initial conditions for the simulations.

**Implementation.** All simulations were performed using a simulation tool implemented in C++ that accounts for cell divisions and that previously has been used for simulating yeast cell colonies and plant tissue (see e.g.[6,12]). The ordinary differential equations were solved numerically using a 4th order Runge-Kutta method [13].

**Parameter values**

| **Parameter** | **Value** | **Description** |
| --- | --- | --- |
| Mechanical parameters | | |
|  | 1.0 | Friction coefficient |
| *k*_cc_ | 1.0 | Strength cell-cell interaction |
| *k*_cw_ | 3.0 | Strength cell-wall interaction |
| *k*_int_ | 1.0 | Strength of internal spring |
| Growth and Division parameters | | |
| *k*_g_ | 1e-5 | Growth rate |
| *L*_Th_ | 2.5, 5, 10, 20 | Cell division length |
| *L*_max_ | 3, 6, 12, 24 | Growth truncation length |
| *p*_div_ | 0.03 | Daughter cell length asymmetry |
| *x*_div_ | 0.001 | Daughter cell coordinate shift |
| Solver parameters | | |
| *h* | 0.5 | Simulation step size |
| *T*_end_ - *T*_start_ | 2.5e6 | Simulation length |

**Supplementary Table P1.** Parameter values used in the simulations. When four values are supplied, they correspond to simulations of different cell aspect ratios (defined as 0.5*L*, *L*, 2*L*, and 4*L* in the text). Smaller values for the step size were also tried, with no effect on the results. The time scale used in the simulation was translated to 0-200 in the presentation of the results.

**References**

1. Groisman A, Lobo C, Cho H, Campbell JK, Dufour YS, et al. (2005) A microfluidic chemostat for experiments with bacterial and yeast cells. Nat Methods 2: 685-689.

2. Andersen JB, Heydorn A, Hentzer M, Eberl L, Geisenberger O, et al. (2001) gfp-based N-acyl homoserine-lactone sensor systems for detection of bacterial communication. Appl Environ Microbiol 67: 575-585.

3. Winzer K, Hardie KR, Burgess N, Doherty N, Kirke D, et al. (2002) LuxS: its role in central metabolism and the in vitro synthesis of 4-hydroxy-5-methyl-3(2H)-furanone. Microbiology 148: 909-922.

4. Dunlap PV, Greenberg EP (1988) Control of Vibrio fischeri lux gene transcription by a cyclic AMP receptor protein-luxR protein regulatory circuit. J Bacteriol 170: 4040-4046.

5. Ozbudak EM, Thattai M, Lim HN, Shraiman BI, Van Oudenaarden A (2004) Multistability in the lactose utilization network of Escherichia coli. Nature 427: 737-740.

6. Jonsson H, Levchenko A (2005) An explicit spatial model of yeast microcolony growth. Multiscale Modeling & Simulation 3: 346-361.

7. Happel Jb, H. (1986) Low Reynolds number hydrodynamics. Dordrecht: Martinus Nijhoff Publishers.

8. Landau LD, Lifschitz EM (1970) Theory of Elasticity. Moscow: Pergamon Pres.

9. Tyson JJ, Novak B (2001) Regulation of the eukaryotic cell cycle: molecular antagonism, hysteresis, and irreversible transitions. J Theor Biol 210: 249-263.

10. Shapiro JA, Hsu C (1989) Escherichia coli K-12 cell-cell interactions seen by time-lapse video. The Journal of Bacteriology 171: 5963-5974.

11. Young KD (2006) The selective value of bacterial shape. Microbiol Mol Biol Rev 70: 660-703.

12. Jonsson H, Heisler MG, Shapiro BE, Meyerowitz EM, Mjolsness E (2006) An auxin-driven polarized transport model for phyllotaxis. PNAS 103: 1633-1638.

13. Press WH, Teukolsky, S. A., Vetterling, W. T., and Flannery, B. P. (1992) Numerical recipes in c: the art of scientific computing. New York: Cambridge University Press.

**List of Supporting Movies**

All of the Supporting Moves can be found at:

<https://jshare.johnshopkins.edu/hcho15/public_html/Sup.movies/PLoSSupportingVideos/>.
